# High-power biofuel cells based on three-dimensional reduced graphene oxide/carbon nanotube micro-arrays

**DOI:** 10.1038/s41378-019-0081-2

**Published:** 2019-09-23

**Authors:** Yin Song, Chunlei Wang

**Affiliations:** Department of Mechanical and Materials Science Engineering, Florida International University, 10555 West Flagler Street, Miami, FL 33174 USA

**Keywords:** Electronic properties and materials, Nanoscale materials

## Abstract

Miniaturized enzymatic biofuel cells (EBFCs) with high cell performance are promising candidates for powering next-generation implantable medical devices. Here, we report a closed-loop theoretical and experimental study on a micro EBFC system based on three-dimensional (3D) carbon micropillar arrays coated with reduced graphene oxide (rGO), carbon nanotubes (CNTs), and a biocatalyst composite. The fabrication process of this system combines the top–down carbon microelectromechanical systems (C-MEMS) technique to fabricate the 3D micropillar array platform and bottom–up electrophoretic deposition (EPD) to deposit the reduced rGO/CNTs/enzyme onto the electrode surface. The Michaelis–Menten constant K_M_ of 2.1 mM for glucose oxidase (GOx) on the rGO/CNTs/GOx bioanode was obtained, which is close to the K_M_ for free GOx. Theoretical modelling of the rGO/CNT-based EBFC system via finite element analysis was conducted to predict the cell performance and efficiency. The experimental results from the developed rGO/CNT-based EBFC showed a maximum power density of 196.04 µW cm^−2^ at 0.61 V, which is approximately twice the maximum power density obtained from the rGO-based EBFC. The experimental power density is noted to be 71.1% of the theoretical value.

## Enzymatic biofuel cells: power for medical devices

A two-step process enables the fabrication of miniaturized enzymatic biofuel cells composed of nanomaterials for possible use as power sources in implantable devices. There is growing need for self-powered implanted medical devices. Enzymatic biofuel cells are attractive for this since they rely on bio-compatible and non-toxic materials. However, improvements in power cell density are needed, and agglomeration of nanomaterials on existing devices limits performance. Now, Yin Song and Chunlei Wang from Florida International University report a new process: top–down photolithography creates a high surface area micropillar array, followed by deposition of reduced graphene oxide, carbon nanotubes and enzymes onto the pillars. A maximum power density of 196.04 µW cm^−2^ at 0.61 V is achieved in the resulting devices, attributed to the combination of nanomaterials and conformal coating of the micropillars.

## Graphical abstract

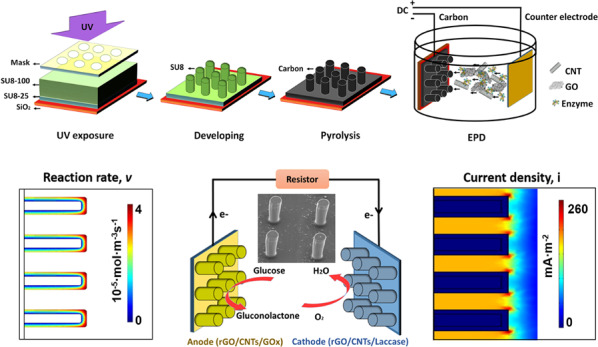

## Introduction

**Fig. 1:**
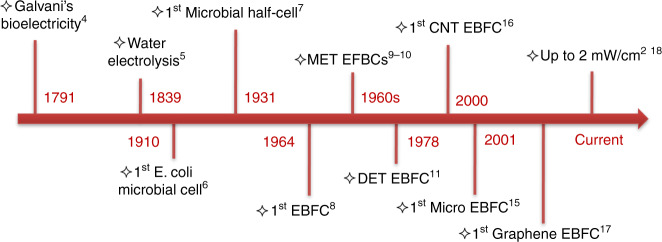
**Milestones of development and utilization of bioelectricity**

Driven by demographic factors such as shifting lifestyle choices, degenerative chronic diseases, and growing geriatric population, the market for implantable medical devices (IMDs) stood at $43.1 billion in 2011 and is expected to increase to $116.3 billion by the end of 2022^1^. Due to economic and ecological concerns, alternative green and efficient power sources should be sought to replace current commercially available lithium-ion batteries. Enzymatic biofuel cells (EBFCs), a subclass of fuel cells that employ enzymes to convert biological energy into electricity, have been touted as a potential power source for IMDs with typical power requirements of micro- to milliwatts^2^. In principle, glucose is catalysed by glucose oxidase (GOx), produces gluconolactone and protons, and generates electrons on the anode. On the cathode, a laccase catalyst reduces molecular oxygen and generates water by combining the oxygen atoms with electrons and protons. EBFCs offer competitive advantages over conventional power sources, including the utilization of renewable and nontoxic biocomponents, high reaction selectivity and activity of biocatalysts, abundance of biofuels, and physiological operating conditions (human body temperature and near neutral pH)^3^. Major milestones in the evolution of bioelectricity generation are illustrated in Fig. 1, i.e., Galvani’s bioelectricity in 1791^4^, water electrolysis in 1839^5^, the initial half-cell using Escherichia coli in 1910^6^, the first microbial biofuel cells in 1931 (later funded by the NASA space program)^7^ and the first EBFC using cell-free enzyme in 1964^8^. The early work on EBFCs in the 1960s involved the use of a purified enzyme and a mediator for performing mediated electron transfer (MET) to the electrode surface^9,10^. Since then, research on EBFCs remained relatively unnoticed until Berezin et al.^11^ made one of the most outstanding contributions by discovering direct electron transfer (DET) in 1978. In the past two decades, few efforts have been made to improve the power density, lifetime, immobilization methods, enzyme loadings, and cell designs^12–14^. In 2001, the first revolutionary micro EBFC utilizing a single carbon fibre as the microelectrode and body fluids as biofuel was demonstrated by Heller^15^, which revealed the feasibility of using EBFCs to power miniaturized IMDs. It is also clear that the current trend of developing high-performance micro EBFCs is highly related to recent progress in nanoscience and nanotechnology. The resulting nanostructured electrodes can enable increased surface area and enzyme loading, provide a favourable confined environment for long-lasting immobilization of enzymes, and facilitate high-efficiency electron transfer while circumventing the need for mediators. The power densities of CNTs and graphene-based EBFCs have already reached a range from a few tens of µW cm^−2^ to almost 2 mWcm^−2^^16,17^, which is sufficient to supply small IMDs. However, challenges remain regarding how to further improve the cell performance of nanomaterials to enable micro EBFCs. Although there has been considerable effort to improve the performance of EBFCs by improving DET, enzyme loading, enzyme lifetime, etc., integrative theoretical and experimental work on combining both aspects of a high-performance electrode material and optimized architecture design is still limited. In particular, carbon nanomaterials such as graphene nanosheets tend to aggregate and restack, and the actual accessible electrode surface areas are much smaller than the theoretical value. In addition, there are many other issues, such as controllability and scalability of contact resistance, interfacial defects, and impurities. Therefore, a scalable process by which high-performance nanomaterials can be effectually integrated onto microstructured electrodes with high surface areas is urgently needed.

Recently, carbon microelectromechanical systems (C-MEMS) and carbon nanoelectromechanical systems (C-NEMS) have been regarded as promising platforms for various electrochemical energy storage, power generation, and biosensing applications, such as lithium-ion batteries, supercapacitors, DNA, and protein biosensors^18–36^. The C-MEMS structures based on pyrolyzed patterned photoresist could also serve as useful platforms for miniaturized EBFCs. The resulting three-dimensional (3D) microstructures with high aspect ratios significantly increased the surface area over limited footprint areas, and the customized current collectors and microelectrodes could be patterned by a straightforward photolithography process on Si wafers. Particularly for on-chip miniaturized EBFC application, C-MEMS/NEMS could provide the opportunity to integrate various semiconductor devices and energy storage/power generation devices on the same Si substrates. In addition, our previous theoretical modelling study of C-MEMS-based EBFCs in both the steady state and transient state revealed general design rules for microelectrode arrays and demonstrated the useful performance of micro EBFCs to power IMDs in a blood artery^37,38^. Recently, the practical integration process of nanomaterials onto C-MEMS microstructures has been developed^29^. The 3D graphene/enzyme composite-based EBFC generated a maximum power density of 136.3 μW cm^−2^ at 0.59 V, which is almost seven times the maximum power density of the bare 3D carbon micropillar array-based EBFC^35^. We also studied one-dimensional (1D) nanomaterial CNTs as spacers between graphene nanosheets as an effective strategy to prevent the aggregation behaviour of 2D materials^38^. The CNTs not only prevent restacking of graphene sheets by acting as nanospacers but also enhance the kinetics of the electrode, thus resulting in a high-frequency response in interdigitated micro-supercapacitors with superior time constants as low as 4.8 ms^36^. In the present research, a remarkable closed-loop work is reported containing both theoretical and experimental studies on C-MEMS-based micro EBFCs with reduced graphene oxide (rGO) (2D)/CNTs (1D) hybrid nanomaterials integrated on 3D carbon micropillar arrays. The fabrication methods of this study combine top–down C-MEMS technology to build 3D micropillar arrays with bottom–up electrophoretic deposition (EPD) to co-deposit enzyme/nanomaterial composites onto 3D micropillar platforms. The reduced graphene oxide/carbon nanotube (rGO/CNT)-based 3D EBFC generated a maximum power density of 196.04 µW cm^−2^ at 0.61 V, which was noted as 71.1% of the modelling result. We believe that the scalable approach reported in this study to fabricate high-performance miniaturized EBFCs will shed light on new strategies for EBFC research.

## Results

### Characterization of bioelectrodes

**Fig. 2:**
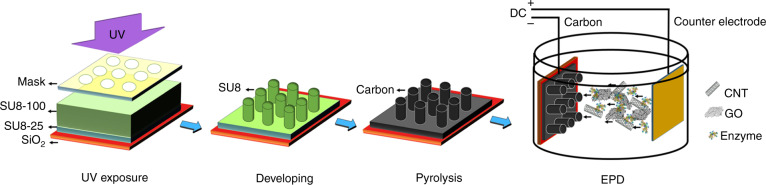
**The fabrication process: top–down C-MEMS to fabricate the 3D micropillar array platform and bottom–up EPD to deposit the rGO/CNTs/enzyme onto the electrode surface (not to scale)**

**Fig. 3:**
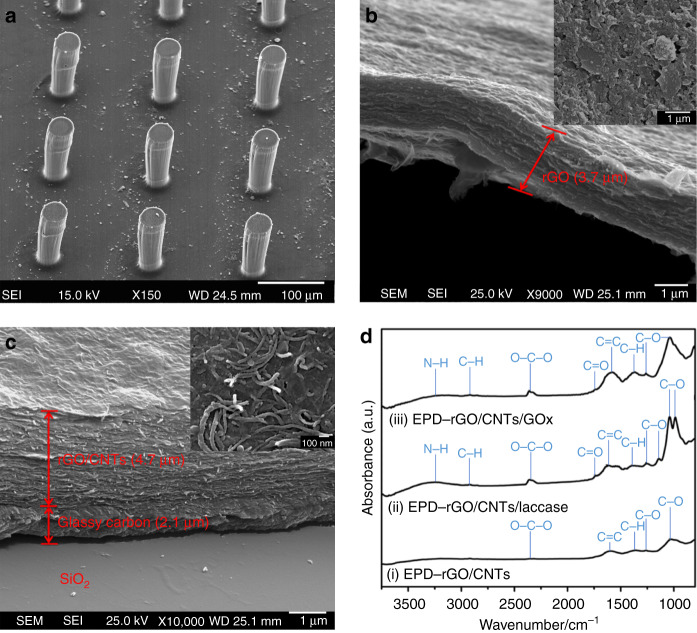
**Characterization of bioelectrodes after EPD.** SEM images showing the morphology of **a** rGO/CNT/GOx-encrusted 3D carbon micropillar arrays; **b** cross-sectional view of thin film and top view of a single micropillar (inset) of rGO/GOx; **c** cross-sectional view of thin film and top view of a single micropillar (inset) of deposited rGO/CNTs/GOx; **d** FTIR spectra of (i) EPD-rGO/CNTs, (ii) EPD-rGO/CNTs/laccase, and (iii) EPD-rGO/CNTs/GOx

The fabrication process of bioelectrodes, including top–down C-MEMS technology and bottom–up EPD, is illustrated in Fig. 2. The 3D micropillar arrays were constructed by the C-MEMS technique^18–20^, which involves a two-step photolithography process followed by a pyrolysis step. Then, EPD was performed to co-deposit enzyme/nanomaterial composites onto the 3D carbon micropillar arrays. The microstructure of EPD co-deposited enzyme/nanomaterial composites onto C-MEMS-based 3D micropillar arrays was investigated by scanning electron microscopy (SEM). As shown in Fig. 3a, the enzyme/nanomaterial composite thin film was seamlessly deposited with good homogeneity on both the conductive 2D current collector and high respect ratio 3D micropillar arrays. The height of a single cylindrical micropillar was ~120 µm, and the diameter was ~35 µm, while the centre-to-centre distance of the closest micropillars was ~130 µm. The cross-sectional images of rGO/GOx and rGO/CNTs/GOx indicated that the thicknesses of nanomaterials/enzyme composite thin films are ~3.7 and 4.7 µm, respectively. In Fig. 3b, a 35° tilted view from the cross section of the rGO/GOx thin film peeled from the C-MEMS 2D current collector layer also showed the local folding and stacking of the rGO layers. Stacked graphene nanosheets can be observed with extended irregular porous structures. In addition, from the top view of the co-deposited rGO/GOx film on the top of one micropillar (inset in Fig. 3b), small stacks of rGO nanosheets with micro-sized wrinkles from GO bending during the EPD process could be observed. As expected, the rGO maintained its fidelity and the firm shape of the stack. Furthermore, pores with dimensions of approximately several hundred nanometres between the stacks of graphene layers were also observed. However, the heavily stacked rGO nanosheets would inhibit the diffusion of mass transport species into the rGO film and hinder the electron transfer efficiency from the enzyme to the electrode. To prevent rGO from restacking, 10 wt% 1D multi-walled CNTs were mixed with 90 wt% 2D rGO, and the mixture was deposited by EPD under the same conditions. The 40° tilted view from the cross section of the rGO/CNTs/GOx thin film on the C-MEMS 2D current collector layer is shown in Fig. 3c. The film clearly showed uniformly packed rGO nanosheets with the appearance of CNTs between the nanosheet layers. The addition of 1D CNTs significantly decreased the rGO restacking and created a 3D network between 2D rGO nanosheets. The unique microstructures and addition of CNTs are expected to facilitate easy penetration of mass transport species and improve electron transfer efficiency between the enzymes and microelectrodes.

Although the oxygenated functional groups in GO can indeed give rise to remarkable structural defects for functionalization, the loss in electrical conductivity could limit the ability of GO as an electrically active material. Thus, the reduction of GO to rGO is highly desired. Analysis of the surface chemistry of the deposited films with Fourier transform infrared spectroscopy (FTIR) indicated that the GO was reduced to rGO during the EPD process. The FTIR spectra of GO before and after deposition are shown in Fig. S1. The broad adsorption peak centred at ~3310 cm^−1^ in the spectrum of GO was assigned as isolated hydroxyl groups^39^. Water, which exhibits signals from O–H bonds at 1600 cm^−1^, was observed^40^. The existence of –O–C–O– bonds was confirmed by the peak at 2340 cm^−1^ ^41^. The peak at 1030 cm^−1^ was consistent with C–O stretching vibrations^40^. The presence of phenol C–O groups and carboxylic acid C = O groups was indicated by the peaks at 1250 cm^−1^ and 1730 cm^−1^, respectively^40,42^. According to the structural model of GO, these functional groups could exist on the periphery of the GO nanosheets. After the EPD process, the intensities of signals from oxygen functionalities were significantly weakened. The spectrum of EPD-rGO exhibited mainly peaks originating from C–O and C = O stretching vibrations.

The immobilization of GOx and laccase on the anode and cathode, respectively, was also studied by FTIR. Figure 3d(i) presents rGO/CNTs on the micropillar, in which there is no obvious characteristic peak. After EPD deposition of rGO/CNTs/laccase on the biocathode, various absorption peaks were observed, as shown in Fig. 3d(ii). The absorption peak centred at ~3250 cm^−1^ was assigned as the N-H stretching vibration, which is a characteristic peak for amino groups from enzymes^41^. The peak at 2340 cm^−1^ was consistent with the O–C–O stretching vibration, also indicating the existence of the enzyme^42^. The peak at 1730 cm^−1^ assigned to C = O stretching vibrations from carboxylic groups and C = C stretching vibrations at 1625 cm^−1^ could be observed as well^40^. The phenolic C–O peak at 1250 cm^−1^ was from carbonyl groups, and the peak at 1030 cm^−1^ was from C–O stretching vibrations^42^. The spectrum also showed the presence of epoxy C–O stretching at ~1000 cm^−1^^42^. The FTIR spectrum for the EPD-deposited rGO/CNTs/GOx bioanode is shown in Fig. 3d(iii). Similar characteristic peaks were observed as well. As is known, the amino groups (–NH_2_) and carboxylic groups (–COOH) are abundant in GOx and laccase. Therefore, the FTIR results clearly indicated the successful immobilization of the enzymes with the rGO/CNT composite on the 3D carbon micropillar arrays. For comparison, the rGO/enzyme without CNTs was also investigated by FTIR, as shown in Fig. S2, and similar surface functionalization and immobilization were achieved.

**Fig. 4:**
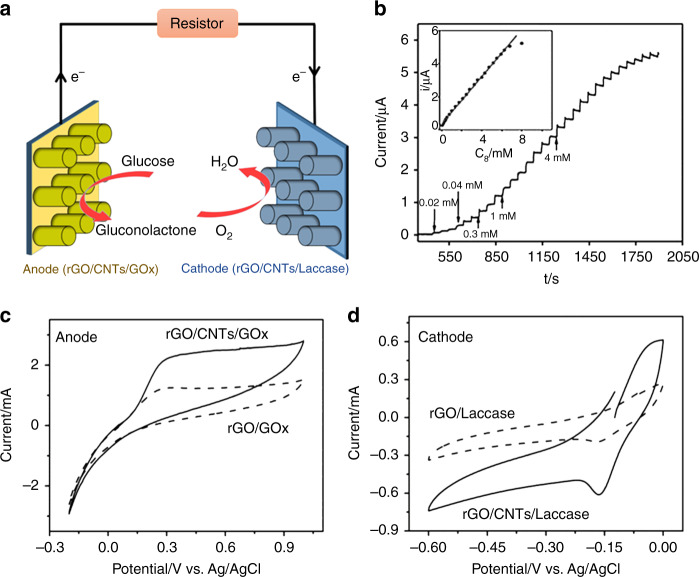
**Electrochemical performance of fabricated bioelectrodes.a** Schematic of an EBFC based on C-MEMS; **b** amperometric response of the rGO/CNTs/GOx bioanode over time at an applied potential of 0.05 V. The inset is an amperometric response upon successive addition of glucose in PBS solution; **c** cyclic voltammograms of the rGO/GOx and rGO/CNTs/GOx bioanodes in 100 mM glucose PBS solution at a scan rate of 50 mV/s; **d** cyclic voltammograms of the rGO/laccase and rGO/CNTs/laccase biocathodes in oxygen-saturated PBS solution at a scan rate of 50 mV/s

The schematic drawing of the EBFC based on C-MEMS is illustrated in Fig. 4a. To calculate the Michaelis–Menten constant (K_M_) of GOx on the developed rGO/CNTs/GOx bioanode, which is relative to the enzymatic affinity and the ratio of the microscopic kinetic constant, the current-time relationship of the rGO/CNTs/GOx bioanode on additions of glucose (from 0.02 to 8 mM) at an applied potential of 0.05 V was investigated. The results showed that the bioanode could respond very rapidly to changes in the glucose concentration (Fig. 4b). The response displayed a linear relationship at glucose concentrations ranging from 0.02 to 7.24 mM. Based on the slope, the K_M_ of GOx after co-deposition was calculated to be 2.1 mM according to the Lineweaver-Burk equation^43^. The resulting K_M_ is higher than that of free enzyme (1.8 mM), which means that GOx is less active after EPD on the microelectrodes. However, the resulting K_M_ is much smaller than the average published K_M_ of GOx using different immobilization methods^44–46^. This result indicates that the enzyme after EPD-based immobilization could remain comparatively active. To compare the electron transfer behaviours of the bioanodes and biocathodes and to verify successful immobilization, cyclic voltammograms were performed. From Fig. 4c, the rGO/GOx and rGO/CNTs/GOx bioanodes exhibited redox peaks at maximum currents of ~1.1 mA and 2.2 mA, respectively. Upon comparison, the rGO/CNTs/GOx bioanode presented a much better redox activity, which can be attributed to a more accessible surface area and more efficient electron transfer. Similarly, the rGO/CNTs/laccase biocathode exhibited a much higher maximum redox current than the rGO/laccase biocathode, as shown in Fig. 4d. The above comparison of both bioanodes and biocathodes suggested that the rGO/CNT/enzyme-based 3D carbon micropillar arrays are more suitable for high-performance EBFCs.

### Simulation of rGO/CNT/enzyme-based EBFCs

**Fig. 5:**
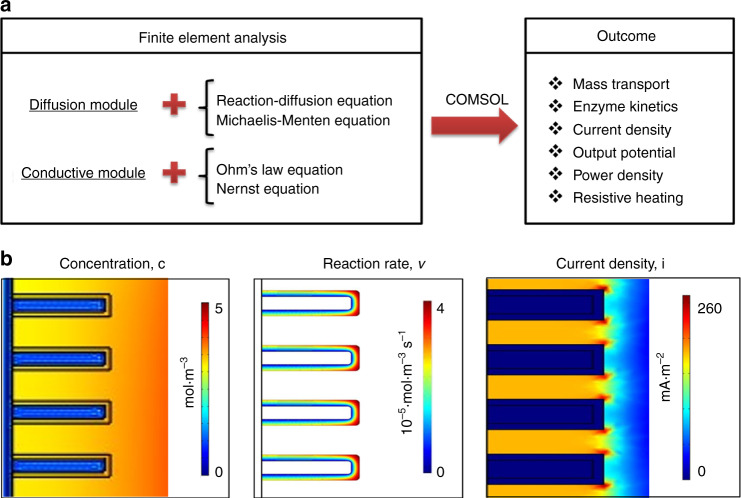
**Simulation results of EBFCs.a** Simulation flow chart; **b** cross-sectional profiles of EBFC in terms of concentration, reaction rate, and current density

**Fig. 6:**
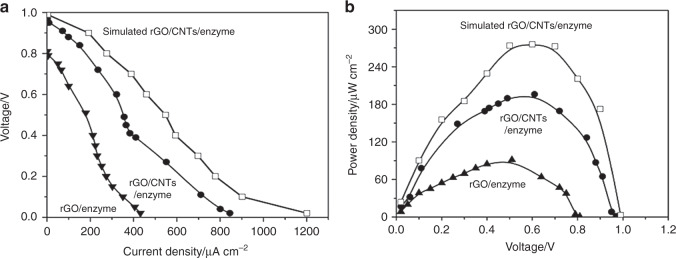
**Cell performance of EBFCs.a** Current density and voltage relationship of the rGO/CNT/enzyme-based EBFC, rGO/enzyme-based EBFC and the simulated rGO/CNT/enzyme-based EBFC; **b** Power density performance of the rGO/CNT/enzyme-based EBFC, rGO/enzyme-based EBFC and the simulated rGO/CNT/enzyme-based EBFC

To predict the performance of the developed rGO/CNT/enzyme-based EBFCs, a detailed modelling of the EBFC system was conducted using COMSOL Multiphysics, which solves partial differential equations by finite element analysis (Fig. 5a). First, mass transport was investigated for the glucose and oxygen diffusion around electrodes in microelectrode arrays. Ideally, glucose should interact with the total surface area of electrodes from top to bottom to fully utilize the immobilized enzymes. However, the glucose reacts immediately with the top portion of the electrode when the molecule approaches the electrode arrays, and the rest of the glucose reacts gradually when it diffuses to the bottom of the electrode. From the concentration profile shown in Figs. 5b and S3, non-uniformity of the glucose concentration along the surface of electrode was observed. There was a gradual decrease in the glucose concentration inside the well from the top to the bottom of the 3D electrode. The competition between a higher enzyme reaction rate and a lower diffusion rate causes glucose depletion throughout the electrode surface and consequently generates a non-uniform glucose concentration. Since the concentration gradient on the surface of the electrode influences the enzyme kinetics defined by the Michaelis–Menten reaction rate, this gradient leads to the observed enzyme reaction variation in the enzyme layer as shown in the reaction rates in Figs. 5b and S4. From the simulation results, the enzyme reaction rate decreased from top to bottom along the surface of the microelectrodes, which is in good agreement with the concentration gradient profile. Moreover, compared with the inner enzyme layer, the outermost surface of the microelectrode exhibited a higher enzyme reaction rate due to diffusion. In addition, the noticeable maximum enzyme reaction rate at the top edge of the microelectrodes resulted from the edge effect. When the substrate reacted with the enzyme immobilized on the electrode, the competition between higher enzyme reaction rate and lower diffusion rate caused a non-uniform glucose concentration and non-uniform reaction rate, consequently leading to a non-uniform current density increasing from the bottom to the top of the electrode. In addition, the change in resistive heating, a process by which the passage of an electric current through an electrode generates heat, is shown in Fig. S5. It is observed that the maximum current density and resistive heating occurred at the top corner of each micropillar electrode. Due to the edge effect, the resistive heating was almost five times higher at the top corners compared with other locations. Various external loads in the range of 0.5–500 kΩ were considered in the simulation to derive the power density-voltage relationship. As shown in Fig. 6, the simulated total current density vs. voltage and voltage vs. power density are plotted. Here, the integration of the current density along the electrode surface has been used to calculate the total current density. The power density of the EBFC increases as the voltage increases, reaching a maximum value of ~272 μW cm^−2^ at 0.58 V, and then decreases with increasing voltage.

### Evaluation of rGO/CNT/enzyme-based EBFCs

Two types of EBFCs were constructed using rGO/enzyme and rGO/CNTs/enzyme bioelectrodes, as schematically shown in Fig. 4a. Cell performance was evaluated by varying the external resistors from 0.5 to 500 kΩ between the bioanode and the biocathode. The current density vs. voltage behaviour of the rGO/enzyme-based 3D EBFC at various external resistors is shown in Fig. 6a. The open-circuit voltage and the maximum current density were found to be 0.81 V and 431.2 µA cm^−2^, respectively. Similarly, the rGO/CNT/enzyme-based 3D EBFCs were constructed and evaluated under the same conditions, resulting in an open-circuit voltage of 0.88 V and a maximum current density of 844 µA cm^−2^. The significant increase in the open-circuit voltage and maximum current density can be attributed to the unique microstructure of the electrode arrays and improved kinetics of the rGO/CNT composite.

Furthermore, the power densities of the two EBFC systems were calculated, and they are plotted in Fig. 6b. The maximum power density of the rGO/CNT/enzyme-based EBFC was calculated to be 196.04 µW cm^−2^ at 0.61 V, which is approximately two times the maximum power density of the rGO/enzyme-based EBFC (91.34 µW cm^−2^ at 0.51 V). By comparing both experimental and theoretical maximum power densities, the efficiency of the experimental rGO/CNT/enzyme-based EBFC reached 71.1% of the maximum simulated value. In addition, the stability of the rGO/CNT/enzyme-based EBFC was evaluated. After 7 days, the maximum power output dropped to 35.5%. After operation, the microstructure of the EPD co-deposited enzyme/nanomaterial composites on C-MEMS-based 3D micropillar arrays was again investigated by SEM. As shown in Figs. S6 and S7, the cross section and surface of rGO/CNTs/GOx with a thickness of 4.6 ± 0.1 µm displayed structural fidelity in terms of morphology and dimension. The decreased performance could be due to the inherent instability of enzymes. To improve the lifetime of EBFCs as power supplies for small electronics, several strategies could be attempted, such as physical trapping of enzymes, substitution of new enzymes or addition of chemicals for increasing stabilities of enzymes^47–49^.

## Discussion

The novelty of this study is to combine 1D and 2D carbon-based materials in the form of 3D carbon microstructures to develop high-performance micro EBFCs. Mano et al.^50^ reported a high-performance EBFC generating an areal power density of 740 µW cm^−2^ using a single carbon nanofibre. Nevertheless, the total power of the system could be a concern due to the limited amount of enzyme on the single carbon nanofibre. The resulting novel structure in this work exhibited the following desirable characteristics: (1) conformal coating of composite materials on 3D microelectrode arrays; (2) high surface area and active surfaces; and (3) feasibility and scalability of embedding biocatalysts. Compared with our previous work on graphene-based EBFCs, the performance of the rGO/CNT-based EBFCs has been greatly improved^35^. The prolonged cell life might result from the more stable immobilization of the components due to the functionality of rGO. From the comparison between the experimental and simulation results, the rGO/CNT-based EBFC reached 71.1% of the maximum theoretical performance. The difference in performance between simulation and experiment may be due to the following reasons. First, even though the EPD is known to form uniform deposited layers on different 2D substrates, electric field-induced non-uniform coating on 3D microelectrodes could affect the actual performance. Thus, the uniform boundary condition in the nanomaterial layer could result in a higher simulated cell performance than the experimental results. Second, the distribution and activity of the enzyme in the rGO/CNT layer is hard to predict during EPD, while the subdomain condition in the simulation assumed a uniform distribution and activity of the enzyme. Third, the diffusion of fuel in the enzyme/rGO/CNT layer is most likely not as ideally uniform as assumed in the simulation, where a constant diffusion coefficient was used. Although the simulation has some inevitable limitations, we have demonstrated that finite element analysis is a very useful tool to predict the performance of EBFCs.

In this work, a high-performance EBFC based on a C-MEMS platform and rGO/CNT composites was fabricated. A maximum power density of 196.04 µW cm^−2^ at 0.61 V and 64.5% power remaining after 7 days were achieved. This EBFC performance is adequate to power low-voltage complementary metal-oxide-semiconductor integrated circuits and disposable biosensor applications. The high cell performance could be attributed to the unique rGO/CNTs integrated with the C-MEMS platform, which can facilitate better charge transport and yield high power density. The power density of the experimental value is noted to be 71.1% of the theoretical value obtained from finite element analysis. Our approach is scalable in terms of enzyme loading and cell performance.

## Materials and methods

### Chemicals

Two negative photoresists, NANO^TM^ SU-8 25 and SU-8 100, as well as SU-8 developer, were purchased from MicroChem Corp. (Westborough, MA) GOx (100 U mg^−1^ solid), laccase (20 U mg^−1^ solid) and glucose were purchased from Sigma-Aldrich and used without further purification. Glucose was prepared in phosphate buffer solution (pH = 7.4). Graphene oxide (0.7–1.2 nm in thickness and 300–800 nm in lateral dimensions) and multi-walled carbon nanotubes (30–50 nm in outer diameter) were purchased from CheapTubes, Inc. (Cambridgeport, VT). All aqueous solutions were prepared in deionized water.

### Instrumentation

SU-8 was deposited by a Headway research^TM^ (Garland, TX) photoresist spinner, and an OAI 800 mask aligner was used for UV exposure. The pyrolysis process was conducted in a Lindberg alumina-tube furnace. The morphology of the microstructures was investigated using a JOEL 6335 FE-scanning electron microscope. FTIR (JASCO FT/IR 4100 spectrometer) was used to analyse the functionalized electrode surface. Cyclic voltammograms were measured by a VMP3 multichannel potentiostat/galvanostat (Princeton Applied Research). The resulting bioanodes and biocathodes were connected to an external circuit for EBFC performance testing using a CHI 660 C workstation. Finite element analysis was performed using COMSOL Multiphysics 4.3b commercial software (license no. 1023246).

### Bioelectrode fabrication

The C-MEMS fabrication procedure is shown in Fig. 2. Briefly, C-MEMS-based 3D micropillar arrays were prepared by a two-step photolithography process followed by a pyrolysis step. In the first step, a 2D round (diameter of 8 mm) pattern was formed as the current collector. The NANO^TM^ SU-8 25 photoresist was spin-coated onto a silicon oxide wafer (4″ in diameter, (1 0 0)-oriented, n-type) at 500 rpm for 12 s and 3000 rpm for 30 s, followed by a soft bake at 65 °C for 3 min and a hard bake at 95 °C for 7 min on a hotplate. The photoresist film was then patterned under a UV exposure dose of 300 mJ cm^−2^, followed by a post-exposure bake at 65 °C for 1 min and then 95 °C for 5 min on a hotplate. The second photolithography step was conducted using NANO^TM^ SU-8 100 photoresist to construct cylindrical micropillar arrays on a patterned thin film current collector. The photoresist was spin-coated at 500 rpm for 12 s and 1500 rpm for 30 s followed by a soft bake at 65 °C for 10 min and a hard bake at 95 °C for 45 min. UV exposure was conducted under a UV exposure dose of 700 mJ cm^−2^. A post-exposure bake was performed at 65 °C for 3 min and 95 °C for 10 min on a hot plate. The sample was then developed using the NANOTM SU-8 developer for 5–10 min, followed by isopropanol rinsing and nitrogen drying. Finally, the microstructures were pyrolyzed at 1000 °C for 1 h in a Lindberg alumina-tube furnace under a constant flow of 500 sccm forming gas (95% nitrogen, 5% hydrogen). EPD was performed to integrate rGO/CNTs and the enzyme composite onto the 3D micropillar arrays (see Fig. 2). rGO/CNTs at a weight ratio of 9:1 (1.5 mg/mL) and GOx or laccase (1.5 mg/mL) were dispersed in deionized water and then sonicated for 1 h to form a homogenous mixture. The rGO/CNTs/enzyme composite was then deposited by EPD with a DC voltage of 10 V at a distance of 2 cm for 3 min. During the EPD process, the evolution of gas bubbles at the cathode was observed because of the water electrolysis, and the deposition occurred at the anode. After the process, the bioelectrodes were dried and kept at 4 °C to prevent denaturation of the enzyme. In addition, rGO/enzyme-based control bioelectrodes without CNTs as additives were prepared in a similar way for comparison purposes.

### EBFC simulation

The overall redox reaction of the EBFC is given by

1 $${\mathrm{Anode:}}\quad {\mathrm{Glucose}}\mathop{\longrightarrow}\limits^{{{\mathrm{GOx}}}}{\mathrm{Gluconolactone}} + {\mathrm{2H}}^{\mathrm{ + }} + {\mathrm{2e}}^ -$$

2 $${\mathrm{Cathode:}}\quad {\mathrm{O}}_{\mathrm{2}} + {\mathrm{4H}}^ + + {\mathrm{4e}} - \mathop{\longrightarrow}\limits^{{{\mathrm{Laccase}}}}{\mathrm{2H}}_{\mathrm{2}}{\mathrm{O}}$$

A number of enzymes were found to be capable of DET with an electrode, including laccase, whose ability to catalyse DET has been demonstrated. In GOx, as with many redox proteins, the redox centre flavin adenine dinucleotide (FAD) is buried within the protein core. When GOx catalyses glucose oxidation, GOx–FAD is reduced to GOx–FADH_2_, which can be oxidized by the electrode back to GOx–FAD, as shown in the following reaction:

3 $${\mathrm{GOx - FAD}} + {\mathrm{Glucose}} \to {\mathrm{GOx - FADH}}_{\mathrm{2}} + {\mathrm{Gluconolactone}}$$

4 $${\mathrm{GOx - FADH}}_{\mathrm{2}} \leftrightarrow {\mathrm{GOx - FAD}} + {\mathrm{2H}}^{\mathrm{ + }} + {\mathrm{2e}}^ -$$

To simplify the modelling, we assume that the enzyme (GOx/laccase) and electrode reactions are coupled by DET. In such a system, the coupled overall process is the redox transformation of the substrates, which can be considered an enzyme-catalysed electrode process. In addition, the following assumptions have been made to simplify the computational model:

- The steady-state response is achieved without considering forced convection.
- The enzyme is uniformly distributed in the enzyme layer.
- Negligible change in heat transfer is assumed between the enzyme layer and electrode interface.
- Temperature distribution around the EBFCs is assumed to be uniform.
- Some interfering reactions, such as hydrogen peroxide inhibition, are neglected for simplicity.
- The DET between enzyme and electrode is assumed to simplify the modelling.

Two modules have been applied: (1) diffusion module to incorporate the mass transport and enzymatic kinetics; and (2) conductive module to integrate the concentration and potential. In the diffusion module, the diffusion of substrate with enzyme kinetics is solved based on reaction-diffusion equations:

5 $$\frac{{\partial c}}{{\partial t}} + \nabla \left( { - D \cdot \nabla c} \right) = v$$

where *c* is the concentration of substrate, *D* is the diffusion coefficient, and *v* is the redox reaction rate defined by equation. The efficiency of utilization of the fuel is directly related to the enzyme kinetics. The Michaelis–Menten kinetics for a single-substrate reaction are considered for the anode and cathode, respectively. The steady-state kinetics of the enzyme reaction (*v*) are expressed by

6 $$v = \frac{{k_{cat}[A]}}{{1 + K_M/[S]}}$$

where *k*_cat_ is the catalytic rate constant and K_M_ is the Michaelis–Menten constant of the enzyme. [*A*] and [*S*] are the concentration of the enzyme and the substrate, respectively.

In the conductive DC module, the governing PDE to calculate the potential is given by Ohm’s law equation:

7 $$J = \sigma E + J^e$$

where *J* is the current density, *σ* is the conductivity of the material, *E* is the electrode potential solved by Eq. [8], and *J*^*e*^ is the external current density.

The electrode potentials (*E*) in EBFCs can be related to the Nernst equation, which arises from potential differences produced by chemical reactions:

8 $$E = E^\circ + \frac{{RT}}{{zF}}{\mathrm{ln}}\left( {\frac{{\left[ {C_{ox}} \right]}}{{\left[ {C_{red}} \right]}}} \right)$$

where *E*^*o*^ is the standard potential, [*C*_*ox*_] and [*C*_*red*_] represent the concentration of the oxidized and reduced species (the original activity can be replaced by low concentration), respectively, *R* is the universal gas constant, *T* is the temperature, *F* is the Faraday’s constant, and *z* is the number of electrons transferred in the cell reaction.

The dimensions used in this simulation are consistent with the experimental design. The boundary conditions and the relevant constants are shown in Tables 1 and 2, respectively.

**Table 1:** Boundary conditions for simulation models

Boundary	Diffusion	Potential
Boundary of bulk domain	$c = c_0$	$n \cdot J = o$
Bulk-enzyme interface	$- n\left( {N_1 - N_2} \right) = 0$	$V = V_0$
Enzyme-electrode interface	$- n\left( {N_1 - N_2} \right) = 0$	$n\left( {J_1 - J_2} \right) = 0$

**Table 2:** Simulation parameters and constants

Parameter/constant	Description	Value	Reference
*R*	Universal gas constant	8.314 J·mol·K^−1^	
*T*	Room temperature	300 K	
*F*	Faraday’s constant	96485 C·mol^−1^	
*D*_glucose_	Diffusion coefficient of glucose	7^−10^ m^2^·s^−1^	^51^
*D*_oxygen_	Diffusion coefficient of oxygen	2.13-(0.0092Ht)·10^−9^ m^2^·s^−1^	^52^
K_M_GOx_	Michaelis–Menten constant for GOx	2.1 mM	*Calculated from experimental results
K_M_laccase_	Michaelis–Menten constant for laccase	3.28 mM	^53^
k_cat_GOx_	Catalytic rate constant of GOx	6.5 S^−1^	^54^
k_cat_laccase_	Catalytic rate constant of laccase	2.69 S^−1^	^53^
*E*^*o*^_*A*_	Reference potential for anode	−0.32 V	^55^
*E*^*o*^_*C*_	Reference potential for cathode	0.585 V	^55^
*σ*_carbon_	Conductivity of glassy carbon	8000 S·m^−1^	^56^
*σ*_substrate_	Conductivity of thin film layer	10,000 S·m^−1^	^56^

## Supplementary information

Supplementary Information
