# Supplementary material for: High-power biofuel cells based on three-dimensional reduced graphene oxide/carbon nanotube micro-arrays
Source: Microsyst Nanoeng. 2019 Sep 23;5:46. doi: 10.1038/s41378-019-0081-2 (PMC6799826; doi:10.1038/s41378-019-0081-2)
Supplement: Supplementary file 1 — Supplementary Information [file 41378_2019_81_MOESM1_ESM.doc]

**Supplementary Materials**

**High-Power Biofuel Cells Based on Three-Dimensional Reduced Graphene Oxide/Carbon Nanotube Micro-Arrays**

Yin Song and Chunlei Wang*

Department of Mechanical and Materials Science Engineering, Florida International University, 10555 W. Flagler Street, Miami, FL 33174, USA

* Correspondence: Chunlei Wang (wangc@fiu.edu)


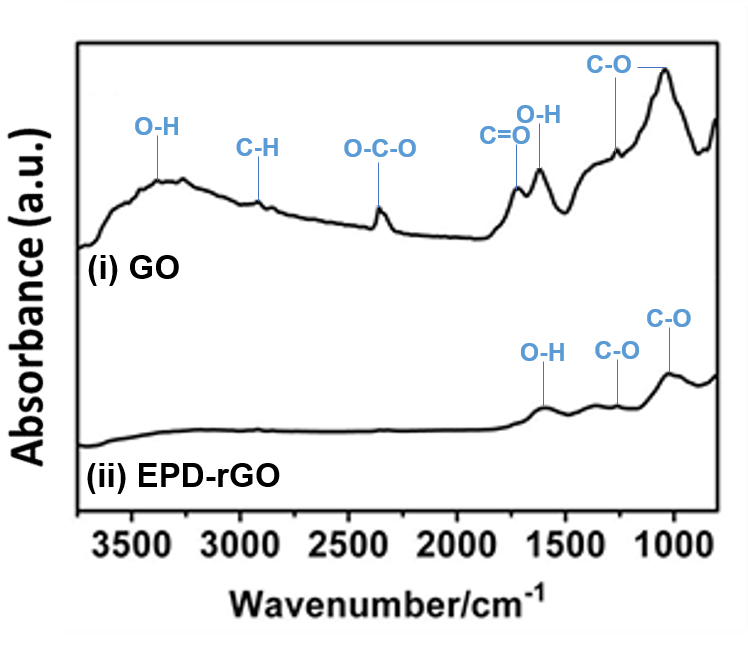


Figure S1. FTIR spectra of (i) graphene oxide (GO) and (ii) electrophoretic deposited reduced graphene oxide (EPD-rGO).


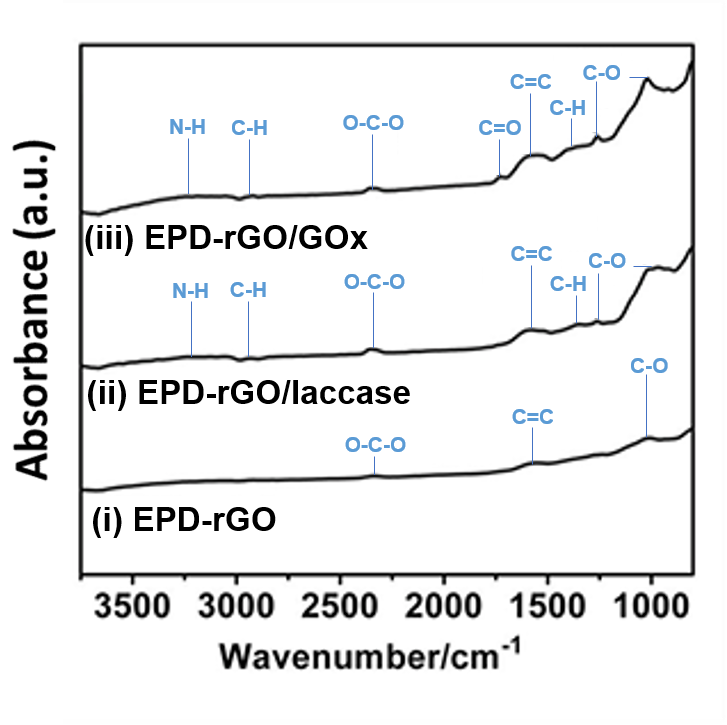


Figure S2. FTIR spectra of (i) EPD-rGO, (ii) EPD-rGO/laccase and (iii) EPD-rGO/GOx.


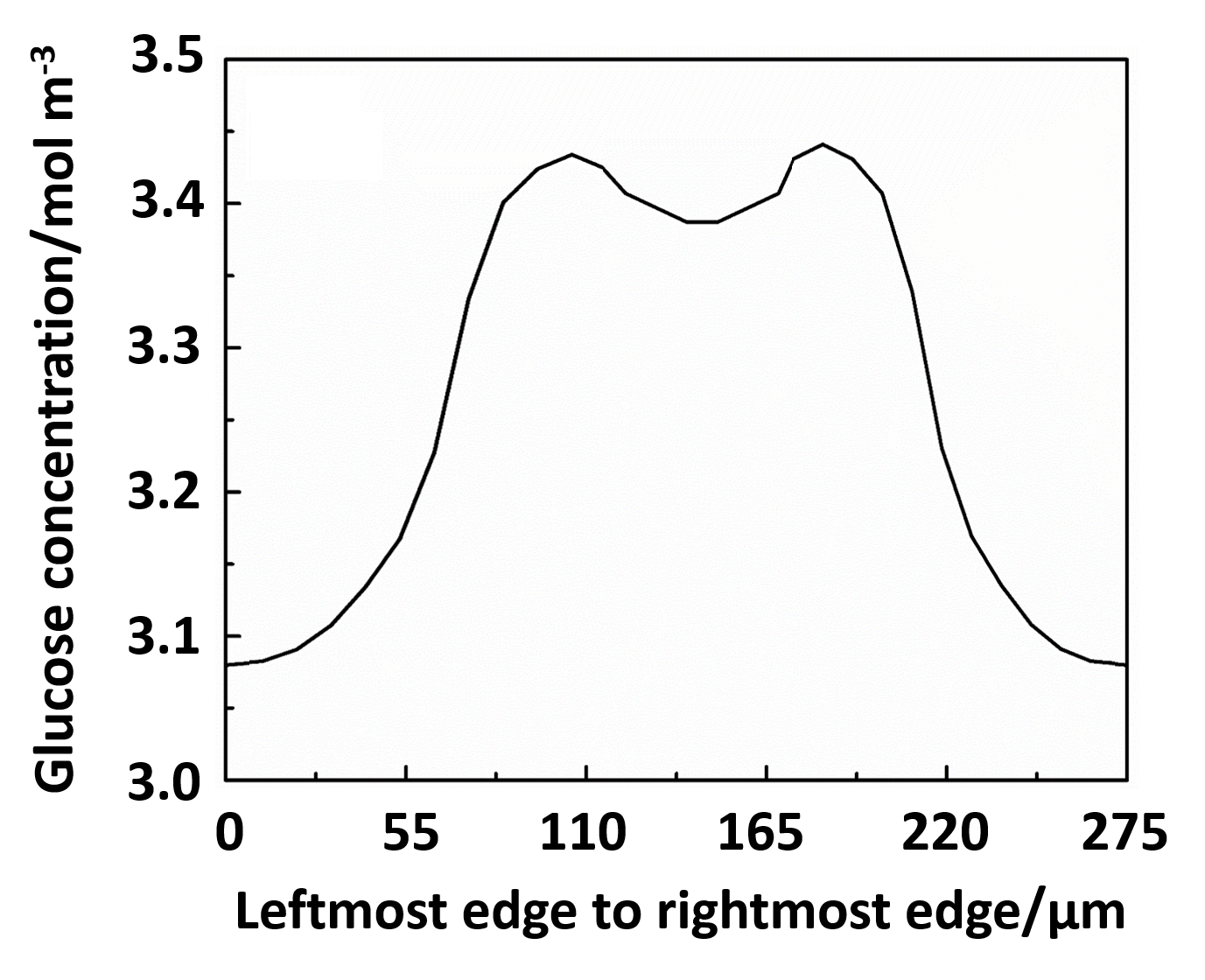


Figure S3. Glucose concentration from both depth wise (0-120 µm, 156-275 µm) and width wise (121-155 µm) along the contour of electrode surface.


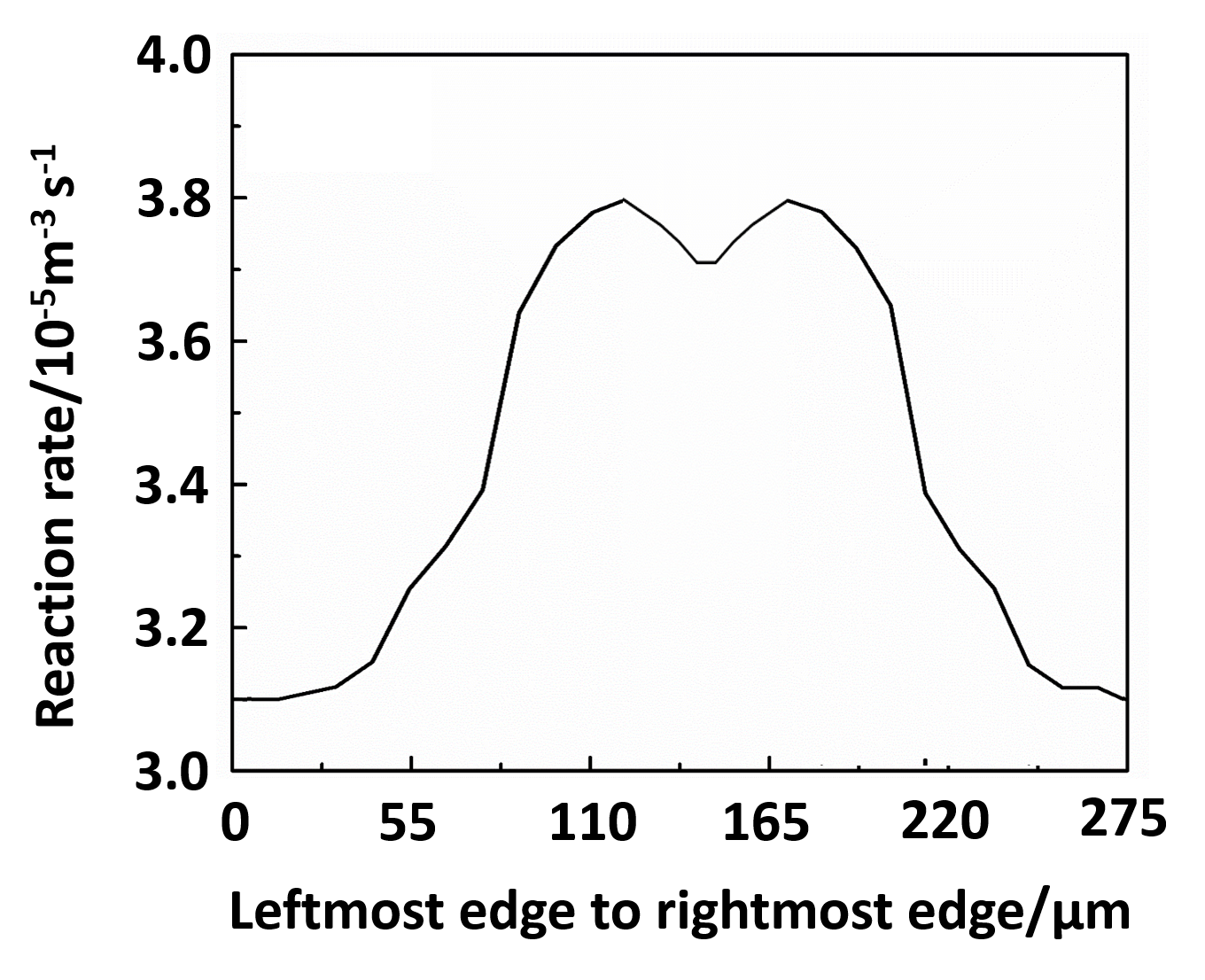


Figure S4. Reaction rate from both depth wise (0-120 µm, 156-275 µm) and width wise (121-155 µm) along the contour of electrode surface.


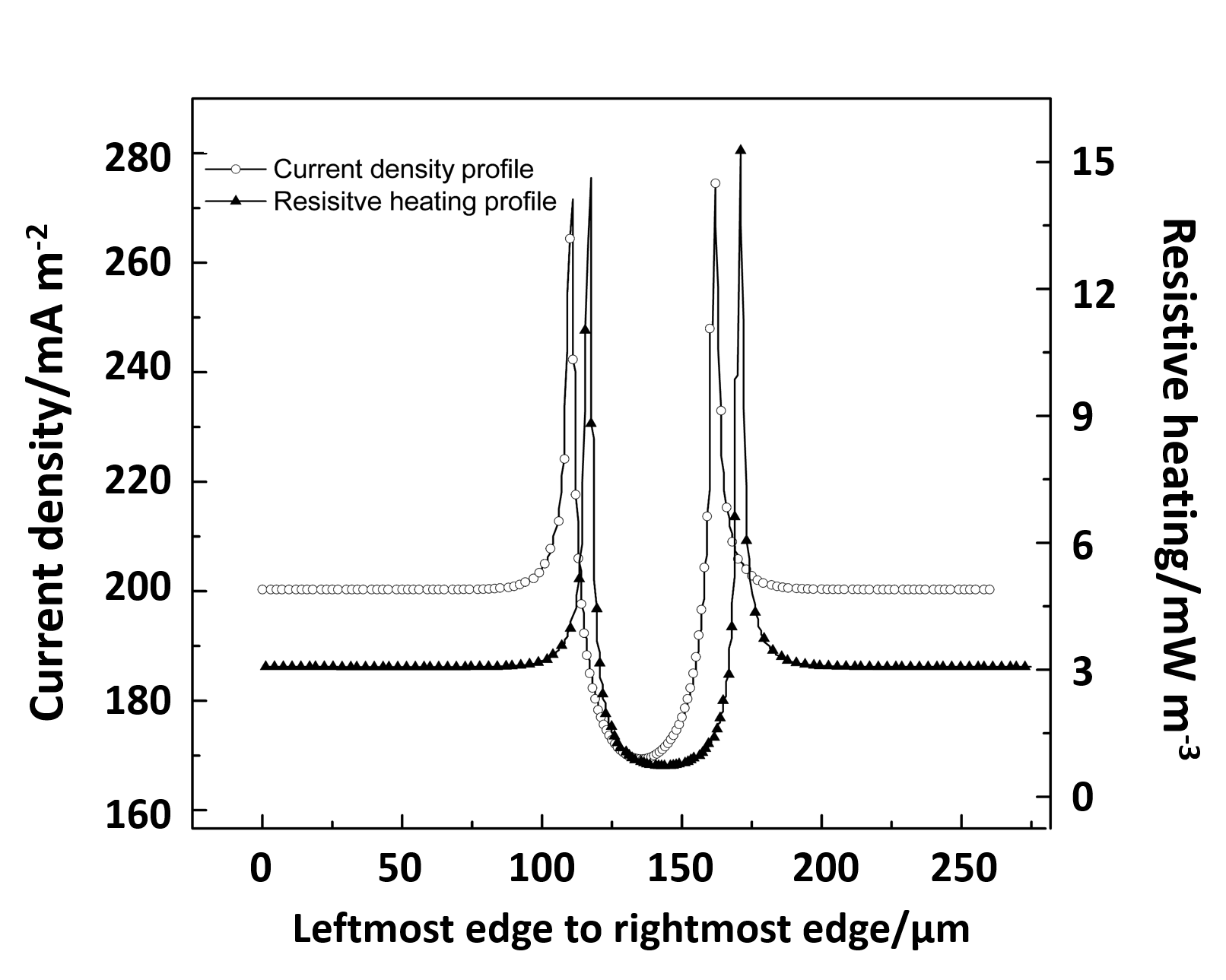


Figure S5. Current density and resistive heating distribution curves from both depth wise (0-120 µm, 156-275 µm) and width wise (121-155 µm) along the contour of electrode surface.


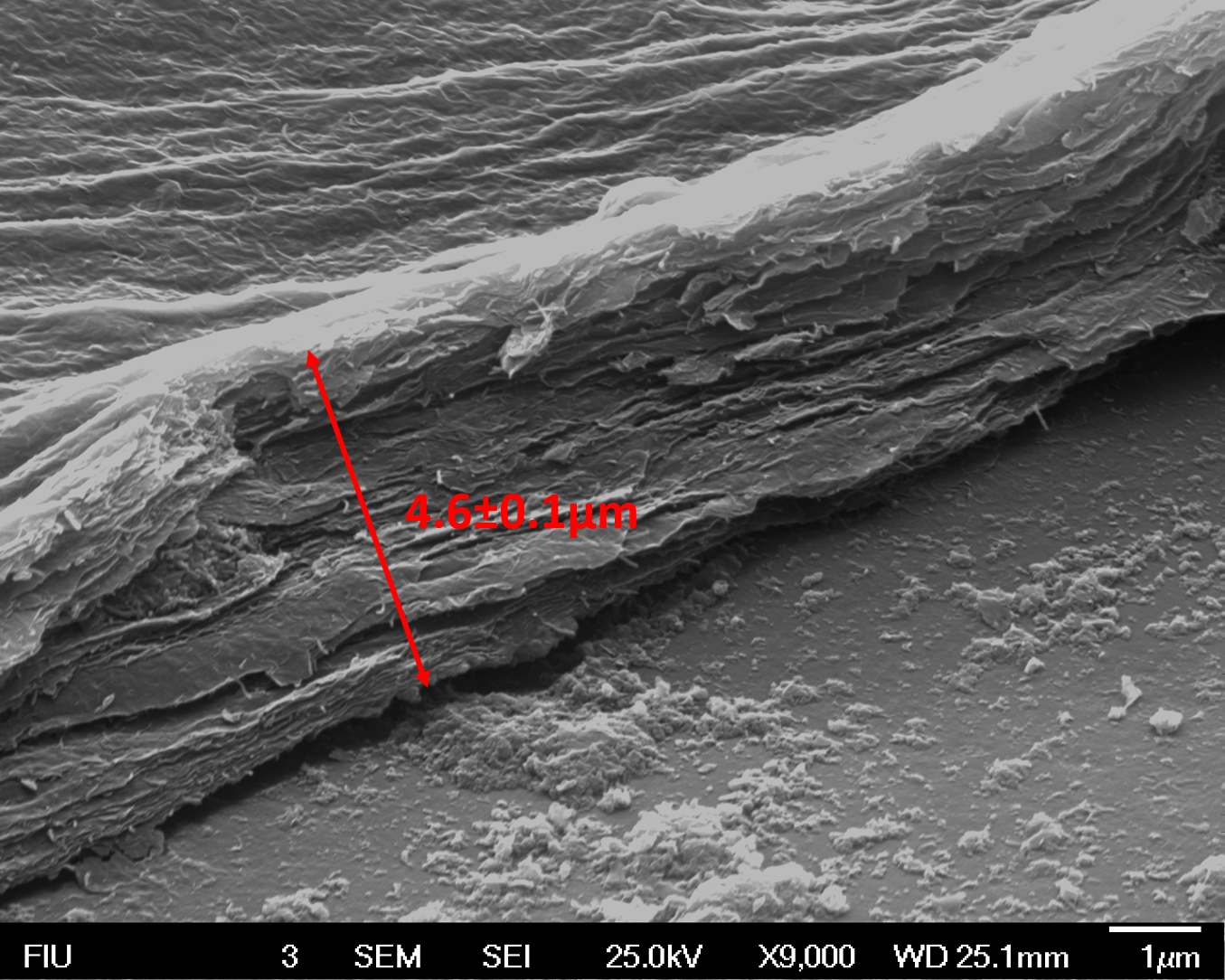


Figure S6. Cross-sectional view of rGO/CNTs/GOx after operation for 7 days.


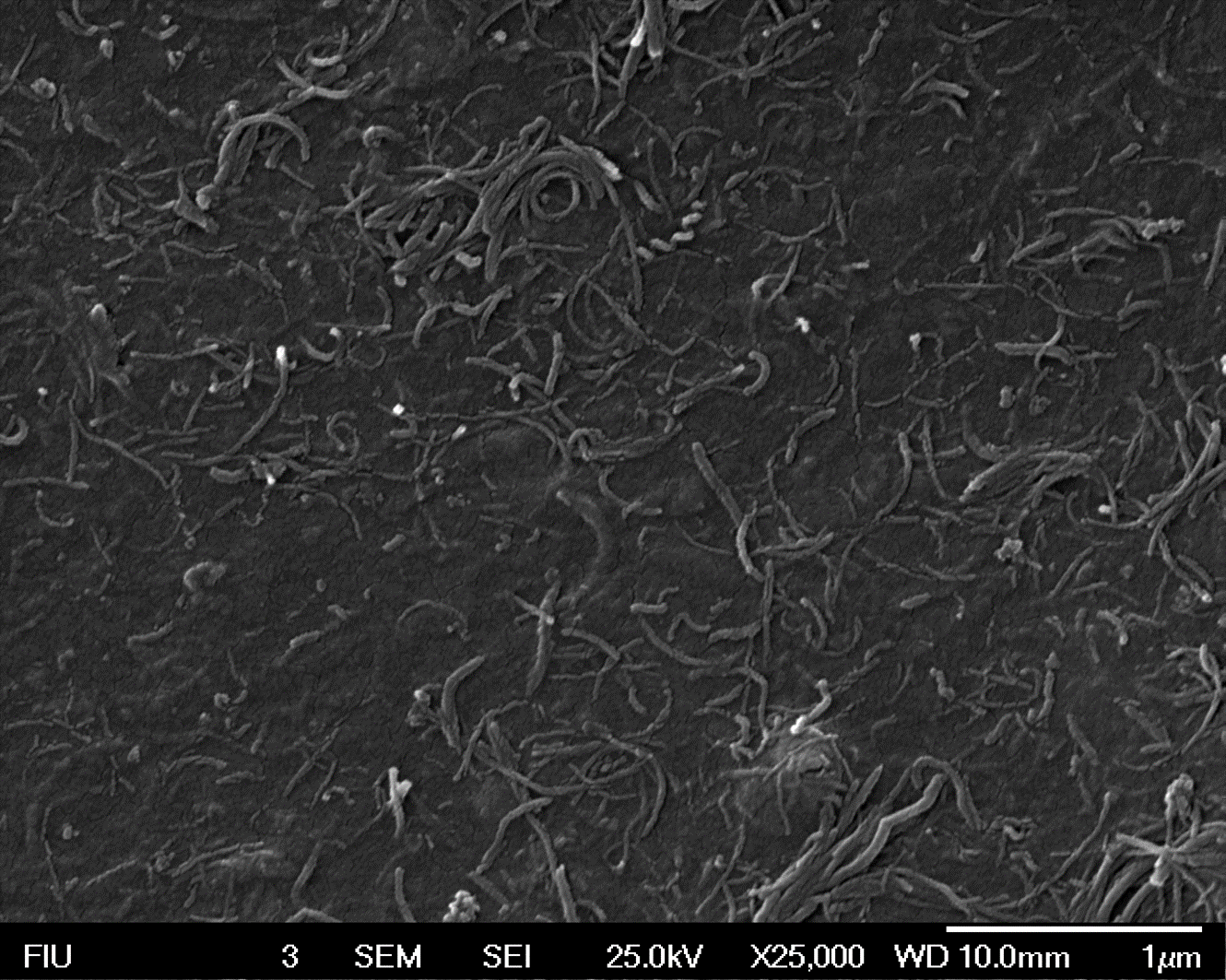


Figure S7. Top view of rGO/CNTs/GOx after operation for 7 days.
